# What do you mean by a periodical? Forms and functions

**DOI:** 10.1098/rsnr.2016.0028

**Published:** 2016-09-21

**Authors:** Pietro Corsi

**Affiliations:** Wellcome Unit for the History of Medicine, University of Oxford, 45–47 Banbury Road, Oxford OX2 6P2, UK

**Keywords:** scientific journals, popular science, science and literature, scientific publishing, nineteenth-century France and England, European circulation of scientific texts

## Abstract

The word ‘periodical’ immediately calls to mind huge stacks of bound volumes neatly arranged on library shelves. Yet, in historical terms, it would be hard to claim that ‘periodical’ is a word endowed with a definite and univocal connotation. Even the criterion of ‘periodicity’ leaves a lot out of account. Forms historically assumed by periodicals often envisaged regular schedules of appearance, but this was often more of a wish than a reality. Thus, great care needs to be taken in accepting at face value the dating of issues and volumes. Scientific periodicals, whether purely professional or purely commercial (and the many forms in between), existed in given historical circumstances and had to compete with different and alternative forms of publication which were also issued in instalments (especially dictionaries and encyclopaedias). They were not the only or even the preferred vehicle for a scientist to communicate or engage in debate. The role of the reading public, in science as well as in many other matters, was never one of a passive receiver: during the long nineteenth century, in several countries, readers claimed the right to intervene in scientific debates, and favoured publications that appeared to accommodate their demands.

Whoever has walked through major library stacks in the Western World, let alone museum libraries, has been stunned by rooms filled up to the ceilings with bound periodicals. Often, much abundant shelf space is also devoted to collections of offprints which famous local savants donated to their institution.^1^ Periodicals are a hard physical fact: a few spanning centuries, several others scores of decades. Volume numbers and years of publications are usually well displayed on the spine, although the title page often refers to successive series of the periodical, indicating breaks and recurring difficulties. Their inescapable materiality is an ostensible testimony to the persistence of scientific communication among communities, often spanning nation states at war with each other.

With the exception of centenary celebrations, scholarship focusing on scientific periodicals as a genre, or as commercial ventures, has taken a long time to develop, even for studies of single titles.^2^ Recently, and the present special issue of *Notes and Records* is only the latest to date, several projects financed by generous research grants are probing the phenomenon of the science journal as a tool of communication, its uncertain past and clouded future.^3^ What appears at first sight as a materially dense, inescapable historical certainty is turning into a multi-level puzzle concerning the procedures of production and the cost of periodicals, the elusive reality of the actual goals pursued, continuity, and forms of readership. We have learned a lot from several pioneering studies that I shall refer to in what follows. As a result, one is tempted to consider the term ‘periodical’ as polysemic as the word ‘press’. Several features attesting the solidity and reliability of the form ‘periodical’ (e.g. the bound volumes, their chronologic span, their function as privileged tools of communication within small or large communities) appear under closer scrutiny to be evanescent to the point of treachery. The preliminary conclusion or methodological lesson to be drawn from the perusal of several in-depth studies would be: ‘Never trust a periodical’; followed by an even more puzzling question: ‘What exactly is or has been a periodical?’ Over the last 10 years or so, English-language scholarship has been very active on the issue of periodicals, to the point that in the following pages I will often and simply add further supporting evidence to conclusions already reached by colleagues. Periodicals are also attracting increasing attention all over Europe, from Portugal to the Urals.^4^ I will myself refer mainly to nineteenth-century publications from countries such as France, Britain, Italy and even Brazil to evoke a global perspective. Three main issues will engage our attention: the considerable growth of the reading public from the late-eighteenth century; the problematic dating of periodical issues and volumes; and the need to consider periodicals within their specific contexts, as one among several competing vehicles for scientific communication and competition.

A major addition to studies on periodicals covering the broader European context, focusing on the period 1650–1800, is the weighty special issue of *Archives internationales d'histoire des sciences*, published in early 2014—though, significantly for our argument, dated June 2013. Twenty-five contributors ambitiously cover an impressive array of case studies, from general culture periodicals to specialized medical or mathematical ones.^5^ In all, 370 periodicals are referred to and placed in their local, disciplinary, economic and national/international context. The conclusion by the editors, Jeanne Peiffer, Maria Conforti and Patrizia Delpiano, is worth quoting in full:

It may even be argued that each and every journal represents a stand-alone story and model – this may appear as a mildly nihilistic stance as regards the need for a common model, and while we are far from endorsing such a view, we have nonetheless reached the conclusion that as regards the relation between science and learned journals no definitive generalization can be provided nor can any contention be safely held, except that periodicals (in the broad spectrum from learned to specialized) did in fact represent a major motivating force in fostering communication, the spread of knowledge and even simple practice in fields such as medicine and mechanics, mathematics, earth sciences, chemical sciences, and antiquarianism. In fact, many journals were so short-lived, or characterized by such a local and limited outreach, that one may doubt whether they did impinge on the creation of a scientific community at a European level, sharing debates, results, and reputations.^6^

The ‘nihilistic’ stand, explicitly denied, but implicitly caressed by our colleagues, stands in sharp and healthy contrast with the opposite temptation, that of seeing an overarching unity within the genre ‘the periodical’. The unifying factor, it has been argued, is the periodicity itself, or, at an epistemological level, the ‘serial’ modality that from the beginning of the nineteenth century marked multiple facets of European economic, social, political and intellectual life.^7^ Personally, I am more inclined towards a nihilist approach, having always found Platonic ideas too sophisticated for my down-to-earth leanings. I would only comment that what a majority of periodicals lacked was precisely chronological consistency, one trait of seriality when publications are concerned. It was announced, boasted about, yet it often remained a Platonic ideal few ventures could aspire to or ever achieve. Periodicals have been born and have died rather quickly, as a statistical norm, from the early seventeenth century to today: well before ‘seriality’ came to represent a ubiquitous heuristic tool to probe the nineteenth century. ‘Serial’ publications and periodicals were on sale since the mid seventeenth century throughout Europe and the colonies.^8^ Moreover, serial displays of Roman and Greek coins, heads of emperors, natural marvels or art of memory *loci* preceded the nineteenth century, and by far. Finally, the rare periodicals spanning centuries are in fact the exception to the rule. Their continuity hides deep discontinuities, and constant financial and identity problems. Long-term survival was due to complex institutional strategies rather than to the intrinsic, explicit function of communication the periodical was supposed to embody. Here, too, the material seriality, the metres of shelf space do not indicate structural and ontological continuity but mere local, institutional, at times contingent capacity to survive.

It is clear that we have collectively taken a few cases of authoritative scientific periodicals that achieved massive impact towards the end of the nineteenth century as the norm throwing light on the past and indicating the way forward. Our reflections on the current crisis of the scientific periodical (from the financial point of view, as an efficient tool of communication, or as an impartial machinery for meritocratic evaluation) may profit from realizing the lesson from history: scientific periodicals (if not periodicals in general) have always been very different from what selective instances from the recent past have led us to believe.

As stated above, in the following sections I will examine a select number of problematic features concerning periodicals, and will briefly refer to examples of periodicals I have actually studied. Through a rather dramatic case of problematic dating and even more problematic authorship attribution, I will stress the often treacherous bibliographic information provided by periodicals. I will then devote a final section to discussion of the first four decades of nineteenth-century French publishing, to highlight the role and secondary function of dictionaries and encyclopaedias as paradoxical forms of commercial periodicals.

## The reading public and its demands

It is safe to generalize that periodicals issued by academies or scientific societies were on the whole less constrained to maintain continuity, and had several strategies to appear to do so, such as sequentially numbering volumes published at irregular intervals. They also enjoyed a relative freedom to stick to their core business (scientific discipline, institutional reporting, etc.). Few ‘academic’ periodicals bothered to discuss Darwin's theories for a long time after the publication of *On the Origin of Species* in 1859. This was impossible for commercial periodicals. They had to convince readers paying by subscription or by instalment that they were doing their best to inform them of national and international news. Thus, when the worsening of the Napoleonic wars made it difficult for periodicals (and everything else Continental) to circulate within the British Isles, academic or scientific society publications did not feel the need to apologize for the lack of updated news or contributions from abroad.^9^ The *Philosophical Magazine* did offer such an apology, even though, significantly, it promised to make up for the lost time.^10^ It is, however, important to stress that perusal of this magazine, as well as of a generous range of periodicals available on Google books, gives the strong impression that the circulation of scientific printed material remained high throughout the Continental Blockade, often through diplomatic channels. It is possible that scientific publications enjoyed a sort of immunity from the embargo, as often scientific personnel or their collections also did. The *Bibliothèque Britannique* of Geneva continued to report on British science even during the French occupation of the Republic and the Continental Blockade. Scientific associations such as the Geological Society of London listed gifts of Continental books throughout the period.^11^

The production and circulation of printed material, and periodicals in particular, varied considerably throughout nineteenth-century Europe. Yet one broad and meaningful generalization is indeed possible. To reach it, one has to reverse the main assumption historians of science almost naturally endorse, that science was produced and circulated through the publications of established and often illustrious institutions. Circulation occurred, inevitably and always (it is often implied), from the top of scientific excellence down to the common reader, a hard-to-assess population that had very different connotations in different countries. Unfortunately, the vast population of practitioners or fans of ‘scientific’ pursuits in most of nineteenth-century Europe has never attracted the sustained attention of historians, since they have been deemed irrelevant to the ‘history of science’. Needless to say, there is no lack of studies of communication in science, especially in English. Less is known of members of the public who took up their pen to write pamphlets, books or letters to the editors and who felt they were entitled to join in debating scientific issues.

Assessing the reading public is not an easy matter. The rates of literacy in the southern states of the Italian Peninsula, for instance, were vastly lower than in France, Germany and the British Isles, let alone in the Netherlands. People likely to buy a book or subscribe to a periodical represented a minute fraction of the population. Yet, from Edinburgh to Palermo and from Lisbon to St Petersburg, sections of the reading public, very different in size and social composition, felt empowered to argue issues in literature and theatre, religion, philosophy, but especially medicine and the natural sciences, irrespective of their actual proficiency in any of the fields they read about. In a classic study of salon sociability and the making of intellectual authority, Antoine Lilty has shown how, towards the close of the eighteenth century, Parisian consumers of daily and weekly printed material felt entitled to express their views unconcerned with the opinion of high society salons and the court. Roger Chartier has famously added to the literature on the intellectual origins of the French Revolution by commenting upon the constitution of a defiant public opinion that felt empowered to pronounce on key political, economic and social issues.^12^

An unrepentant admirer of the Revolution, Henri-Marie Beyle (1783–1842), better known as Stendhal, wrote in the 1820s, for the British public, a series of fascinating social analyses of the Parisian educated public that lived and at times prospered during the 1810s and the 1820s, stressing their role (actual or imagined) as final arbiters of events political as well as intellectual.^13^ He calculated that about 20 000 Parisian authors needed to keep abreast of anything and everything that constituted public debate, from German philosophy and royal gossip, to the return of the Jesuits.^14^ He compared the growing role of reading rooms and circulating libraries in the British Isles and in France, and agreed that both nations excelled in the diffusion of literary news.^15^ The main difference was that Great Britain had several major and minor centres of literary sociability, whereas in France nothing happened—he claimed—outside Paris. Still, gentlemen of the provinces bought books and periodicals, not to feel too provincial and cut off. Mimicry, he implied, played a crucial role in high intellectual and social circles, real or imagined.^16^

The often hilarious testimony offered by Stendhal and the acute historical analyses offered by Lilty and Chartier, among others, also apply to what we anachronistically call ‘science’, certainly to several of its components, and medicine. As in politics and literary matters, the variously composed reading public was convinced that debating scientific issues was part of their claim to genteel status. Radicals famously appropriated this attitude, and in several countries ‘the march of the intellect’ was to them the lever to pull down the hollow shells of aristocratic and priestly power. Yet, more often than not, polite curiosity did not differ much from the rabid political usage of very similar content. Jean-Paul Marat's (1743–1793) fiery denunciation to the effect that his own theories of light and fire were repressed by the monopolistic, aristocratic Académie did not deter the aristocratic Bernard Germain Lacépède (1756–1825) from writing on similar topics, and holding not dissimilar views: as did Jean-Baptiste Lamarck (1744–1829), and a dozen other major and minor contemporary figures.^17^

The ‘homme de lettres’, however, was not a French invention, even though Paris remained for several decades the hunting ground for successful penmen in search of a career. As Maria Conforti has pointed out, even at the tip of the Italian Peninsula, provincial doctors or amateurs wrote letters to periodicals, and subscribed to them, in order to proclaim their meritocratic title to social standing.^18^ Non-academic journals, reporting on a wide spectrum of cultural affairs (politics was too dangerous to be discussed in the early nineteenth century), spread throughout the Continent, imitating each other, taking in turn *The Spectator* and *The Monthly Repository* or the *Magasin encyclopédique* and the *Revue des deux Mondes* or the great Scottish and English quarterlies as their models, adapting them to local social and economic conditions.^19^

‘Official’ academic periodicals (albeit not all) could afford to be both late and expensive, and did not care much about increasing their circulation. They often (but not exclusively) represented narrow disciplinary concerns and made no mystery of their claim to monopolistic authority. Commercial periodicals had to be more or less on time, had to circulate, to appear open and friendly to the reader, and strike alliances with other publications at home and abroad.^20^ Reporting on scientific issues was part of the offer. Many publications active in the first half of the nineteenth century did not need the ascent of the scientific journalist of the later nineteenth century to fight for their share of an already booming market of scientific news.^21^

Slowly but surely the pretence of part of the reading public of pronouncing on scientific matters was reduced by the establishment of specialized disciplinary jargons and publications. The amateur aspiring savants in the novel *Bouvard et Pécuchet* (1881) created by Gustave Flaubert (1821–1880) are a fictional case in point. The social and epistemological process we call ‘specialization’ was well under way, and still continues today. By the early decades of the twentieth century there was little doubt as to what constituted an authoritative scientific journal, ostensibly driven by rules of transparency and collective responsibility. After the Second World War, Anglo-American periodicals and their modes of operation appeared for a time as the natural point of arrival of a long, linear process of development in scientific communication. Yet, one just needs to peruse the vast literature produced by the latest centenary of Darwin in 2009, or remember the hot debates on sociobiology, and the on-going ones on genetic determinism, to witness the extent to which the reading public feels entitled—and rightly so, in many cases—to express their views, and the extent to which scientists presenting themselves as the depository of a superior, technical know-how are at times part of the same reading public when they draw unwarranted conclusions inspired by shared ideology.

Throughout the nineteenth century, the divide between specialized and public knowledge was constantly shifting, discipline by discipline, country by country, but on the whole it was never as marked and accepted as scientists hoped it would be.^22^ To take a telling instance, mathematical gimmicks to entertain fashionable friends or the application of probability to card games were cultivated by aristocratic circles in eighteenth-century France. The sophistication of mathematical thinking in the second half of the nineteenth century made the discipline less attractive to members of the aristocratic salons portrayed by Marcel Proust (1871–1922). Theories of the earth and the universe, of the origin of life, evolution, the so-called ‘science of race’ continued to attract widespread attention and provided material for endless discussions in the daily and the periodical press. On the whole, during much of the nineteenth century, the reading public shared the same social and intellectual leanings as the academic scientist: our contemporary hierarchies between popular books and journals, daily publications, public lecturing or textbooks on the one hand, and elite institutional scientific publications on the other, is often artificial and always reductive. Indeed, during much of the nineteenth century, contemporaries knew the difference between one genre and the next, but tended to take advantage of them all when suitable, both as readers and as authors.

## The illusion of the clockwork journal

Journals that could not count on the financial support of an institution had to present themselves as at least minimally up to date with events in the rest of Europe. To do so, many resorted to unacknowledged imports from the foreign press. This was a long-standing practice, as the reciprocal generous borrowing between the *Philosophical Transactions* and the *Journal des savants* notoriously demonstrated. To answer the demand for news, prestigious nineteenth-century periodicals (scientific and generalist) continued the practice of borrowing, which, I hasten to say, nobody objected to. This is an often-neglected feature of the periodical press, and one that at times renders life very difficult indeed for the historian. Scholars have to make sense of seemingly unambiguous bibliographic data that do not stand up to close scrutiny. And they employ critical skills in establishing the authorship of key texts that may not have an author at all, in the ordinary sense of the word. Two issues are here at stake: the problematic reliability of volume and issue dating, and the origin/authorship of the printed texts. For many publications, especially the institutional ones, it would be prudent never to trust the year and issue number displayed on the cover. This is of course a very serious claim. The practical consequence of such a precautionary measure for our daily work is potentially paralysing. A single case involving Scotland and France during the late 1820s will serve to illustrate the point.

As historians of pre-Darwinian debates on life's possible transformations well know, in the early spring of 1829 the *Edinburgh New Philosophical Journal* published an anonymous account of a paper by Étienne Geoffroy Saint-Hilaire (1772–1844), ‘On the Continuity of the Animal Kingdom by means of Generation, from the first Ages of the World to the present Times’. The original French text was ostensibly published in volume 17 of the *Mémoires du Muséum national d'Histoire naturelle*, dated 1828, pp. 208–229. In fact, internal and external evidence suggests that it was written not earlier than 8 December 1828, that is, at the end of the year. A copy of volume 17 of the French *Mémoires*, held in the library of the Natural History Museum in London, bears a manuscript annotation (seemingly by a librarian) on the title page, specifying that instalment No. 1 was out, or received, in October 1828, and the five remaining ones reached the library in 1829 (actual dates were not recorded). Box AJ/15/834 at the French Archives nationales (Pierrefitte-sur-Seine), a nightmarish collection of hundreds of loose papers and folded A3 size folios, helps to solve the mystery. Volume 17 was late, texts were coming in slowly; thus, most of the instalments for 1828 were published in 1829. Precedence was simply given to what was ready for the press: Geoffroy's piece clearly was early in 1829, probably by March, as discussed below. The instalment containing his famous memoir, the fourth for the year 1828, was distributed to the professors of the Muséum on 25 April 1829. As Bill Jenkins has pointed out, on the same day, 25 April, Jameson read to the Wernerian Society a note bearing the same title as the *Edinburgh New Philosophical Journal* article and Geoffroy's memoir*.* Clearly, the coincidence is purely accidental. Volume 17 could not have reached Edinburgh on the same day. And the April issue of the *Edinburgh New Philosophical Journal* was already on sale by 15 April. Yet, we do know what actually did reach Edinburgh early in April 1829, and started the public discussion.^23^

Let us first take a step backwards, and recall that the authorship of the Edinburgh article has aroused considerable scholarly attention. Adrian Desmond has suggested that the author must have been Robert Edmond Grant (1793–1874), the famous Edinburgh ‘Lamarckian’. Recently, a fair amount of agreement appears to be emerging that the author was in fact Robert Jameson (1775–1854), the supercilious professor of natural history at Edinburgh University. James Secord has argued that Jameson could also have been the author of an equally famous 1826 article first attributed to Grant by the old edition of the *Dictionary of National Biography*. Jameson was probably the author of the 1829 text as well. Bill Jenkins, after suggesting that on 25 April 1829 Jameson gave a paper on the same topic, concluded that the aloof professor was definitely the author of the article in the *Edinburgh New Philosophical Journal*.^24^

It is not my intention to discuss here the textual evidence (scant to say the least) allowing the labelling of Jameson as a fellow traveller of the ‘pro-organic change’ camp, or the meaning we attribute to the term ‘Lamarckian’ when applied to such an enigmatic figure as Grant. Jameson had long been aware of French theorizing on changes life might have undergone, and never wrote a word on the subject. After reading again and again all that Grant did publish up to 1840, I find it difficult to declare him a straightforward Lamarckian. In the course of the comparative anatomy he published in the radical *Lancet* (in instalments), where professions of radical science would have been in tune, reference to Lamarck was extremely scarce and uncommitted.

Whatever the state of scholarship on the authorship of the 1829 article, one thing is clear: it was not written by Jameson, Grant or anyone in Edinburgh or England. The article is a word-for-word translation of a short entry that appeared in the French thrice-weekly *Le Globe* (1824–1830) on 1 April 1829, summing up a memoir Geoffroy had read to the Académie des sciences on 23 March 1829.^25^ *Le Globe* had evidently reached Edinburgh before 25 April. The sequence of events is now clear in its broad outline. It is fair to suppose that once he had completed or printed the *Mémoires* article, Geoffroy tried to publicize it by reading a summary to the first available Académie meeting, a venue that attracted French and European press interest. Sure enough, *Le Globe* printed the summary: one Belgian medical periodical and the Edinburgh journal took it up, word for word.^26^

Playing on different tables at the same time was for Geoffroy a common practice during the 1820s and the 1830s. Indeed, the original *Globe* article reads very much like one of the written summaries of his own oral interventions Geoffroy liked to leak to the press and hand out to colleagues. What is revealing is the quickness of the uptake in Edinburgh. It is furthermore interesting to point out that *Le Globe*, by then an opposition, Saint-Simonian publication, was read in Great Britain by a diverse cohort of personalities, including the theologian and logician, Richard Whately, at Oxford.

In the early nineteenth century, as for a few centuries before and afterwards, a European intellectual space did exist, and ideas as commodities conveyed by journals, which were relatively cheap and fast to print and distribute, freely circulated to shock or thrill readers. Since the mid seventeenth century, periodicals freely made use of material taken from foreign publications. At least from the very late decades of the eighteenth century, several periodicals, especially those supported by subscriptions, advertisements and the books reviewed (which were then sold to the readership) turned a necessity into a selling point. Filling pages on time for the next issue made translations and borrowings inevitable, and even something to boast about. In 1818, the *Journal complémentaire du Dictionnaire des sciences médicales* (on which more below) opened with an editorial informing subscribers of the coverage it offered of leading European medical and natural history periodicals. Of the 42 journals listed, 30 were published in German, six in Italian, five in English and one in Dutch. Together with the *Dictionnaire des sciences médicales*, of which the periodical was a clever marketing spin-off, the journal offers an unrivalled, albeit ignored, coverage of biomedical, nature-historical and institutional developments, especially within the German-speaking countries. That the *Dictionnaire* and the *Journal* were widely known throughout Europe, including the British Isles, should solicit further thorough attempts at recreating the European intellectual space I alluded to.

The conclusion for this section is pretty straightforward: never trust dates, and always look at the wider context. For a long time, Europe had been, and was destined to remain, a bitter and merciless battlefield. Yet, ‘virtuosi’, ‘hommes de lettres’, ‘curiosi’, doctors, chemists or mineralogists, and naturalists continued to exchange with little interruption. Private intercourse and epistolary communications could break down for years, but printed material appeared to circulate, overcoming nearly all difficulties.

## The function of periodicals: competitors and allies

As I concluded in the previous section, the neatly packaged volumes filling shelves from floor to ceiling often conceal doubtful information even at the trivial level of dating, and are at times silent on the origin of the information provided.

If communication of scientific information and the diffusion of claims to authority were among the explicit and implicit goals of scientific periodicals, they were not alone in pursuing the same objectives. ‘Scientists’ were the first to consider several options outside academic and institutional periodicals. Scientific personnel, however employed or socially placed, were well aware of the often-haphazard fortunes of institutional publications, and chose among a variety of alternative and parallel venues to reach an audience. When under threat, it was unwise to wait for the next ‘official’, authoritative journal issue to appear.

Before providing an interesting example of this pragmatic attitude we often collectively overlook, let me simply remind us of the fact that one of the ways to avoid long delays, unsatisfactory distribution and low circulation numbers was the systematic use of offprints. The point has already been stressed, and I simply wish to reiterate what I alluded to at the outset, that several European libraries preserve important collections of offprints assembled by a succession of individuals. The offprint was a very effective solution to the problem of circulation. The offprint allowed scholars to create their own circulation lists and authority networks. Not that this always worked, as Gregor Mendel (1822–1884) discovered to his cost: few people paid any attention to the offprint he had distributed. Yet, rather than simply hope your text will be seen by someone, someday, there was nothing more efficient than to dispatch it yourself, with a manuscript dedication and a letter. The ultimate strategy was to hand-deliver the offprint during a trip undertaken to consolidate one's network. Special binding in expensively decorated leather was reserved for presentations to princes and notables of high standing.^27^

A further point needs brief mention. A few institutional scientific periodicals did not make any money at all, running at a loss. In some cases, they had little to communicate of real importance, especially at the outset. The *Bollettino del Reale Comitato Geologico d'Italia*, the periodical of the Geological Survey of Italy launched in 1870, appeared before a single field geologist had been appointed, or a budget and proper offices established. The earliest issues contained articles collected among friends, and with some difficulty. The founder of the *Bollettino*, Igino Cocchi (1827–1913), who had lived in Paris and London for a total of three years, was familiar with the workings of the Société géologique de France and the Geological Society of London. He learned with bitter disappointment that colleagues in Europe doubted whether Italian geologists could do any serious work at all, lacking books, periodicals and maps. Cocchi had the best idea of his otherwise opaque career. The *Bollettino* was launched in order to obtain in exchange sister publications from throughout the world, books to review and maps to comment upon. Cocchi convinced a reluctant Parliament to finance the cost, arguing for the considerable savings the *Bollettino* earned the Survey. Postal costs were covered by the central administration, and foreign distribution ensured by the diplomatic service. The trick worked, the *Bollettino* is still in existence (though, typically, its history, under changing titles, has been far from linear), and the historic library of the Geological Survey of Italy is indeed impressive. To his own Institute of Geology, at Florence University, Cocchi donated 253 thick volumes binding together thousands of offprints. The collection was a monument to his career, he hoped, attesting to the hundreds of correspondents he had gathered, who had dedicated their offprints to him: in vain, since Italian geologists of today, and historians with them, hardly remember his name.^28^

This form of pragmatic printing, serving communication as well as saving expenditure on foreign books and periodicals, has been repeatedly adopted throughout the Western World. As Fabiano Ardigó has recently shown for the Museum of Curitiba (in the far-from central capital of the State of Paraná, Brazil), during the late 1940s a handful of officers there decided to print a periodical. They offered information on Brazilian bees and fossils (the specialty of two naturalists active in the region) in exchange for books and periodicals the Museum could not afford. The Museum Library still bears witness to the initial success of the initiative, though the periodical is long since forgotten.^29^

As stated above, the purpose of this final section is to explore ways in which scientific personnel pragmatically used a variety of publishing media at their disposal in order to inform, argue or defend themselves. It is not my intention to suggest that what occurred in France during the 1820s and the 1830s, the source of my last examples, is typical or normative. The thesis is exactly the opposite. Publications and their circulation never occurred at a categorical level—institutional or scientific periodicals versus the generalist press or the monograph—but each form co-existed in a given social, intellectual and political space. People acted according to their judgement, and at times even against it. Georges Cuvier (1769–1832) famously disliked dictionaries, even though he was supposed to run one together with his brother Frédéric (1773–1838), and argued for a strictly institutional production and circulation of knowledge. Yet in 1825, under fire from a variety of sources, he chose the article ‘Nature’, printed in the spring of that year in the *Dictionnaire des sciences naturelles* (1804–1806, fresh start, 1816–1830) which he only nominally directed, to launch a powerful counterblast. There, he accused *Naturphilosophen*, transformists, philosophical anatomists and supporters of the doctrine of embryological recapitulation, of being deists if not worse.^30^ This was a dangerous accusation at a time when Parliament was debating extreme anti-blasphemy legislation.

Cuvier had direct and indirect control over several institutional and commercial publications. The choice of the dictionary was thus dictated by his realistic assessment of the French situation: he hoped to reach cultivated readers who would not access institutional publications. We have seen that, since the last decades of the eighteenth century, the claim by the Académie des sciences to be the only tribunal to validate contemporary science was constantly challenged in a broad range of publications. A series of major editorial ventures capitalized on the wish of sizeable sections of the reading public to be involved and even to vote with their purses.^31^ From around 1800 to the end of the century, the daily press, weekly generalist journals and medical publications in particular constantly reported on scientific affairs and institutional science. The *Journal de Physique*, the *Magasin encyclopédique*, the *Revue encyclopédique*, the *Gazette médicale* and also journals entirely and exclusively devoted to reporting academic affairs, such as the *Bulletin général et universel des annonces et des nouvelles scientifiques* (1826–1831), edited by the baron André Étienne d'Audebard de Férussac (1786–1836), *L'Institut (*1833–1872), written by Eugène Arnoult (18?–1873), or *L’Écho du monde savant* (1834–1846), mainly edited by Nérée Boubée (1806–1862), offered weekly summaries of, and often long excerpts from, papers read at one of the Parisian academies or scientific societies. Dailies and weeklies, including *Le Moniteur universel* or *Le Globe*, added their share and reported on an array of books and events. After 1835, the *Comptes rendus hebdomadaires des séances de l'Académie des sciences* tried to oppose unauthorized reporting, without much success.

A specific feature of the Parisian publishing scene was the large number of dictionaries and encyclopaedias produced and sold. Some enjoyed European and indeed worldwide circulation: the French language was still the new Latin. In spite of recurring financial crisis and bankruptcies, the French press continued to provide living rooms and affluent readers from Chile to Russia with collective works of Voltaire or Rousseau. As noted above, according to Stendhal, sales of Voltaire's works totalled two million volumes in the decade 1817–1826. Without entering into a critical assessment of figures, there is no doubt that French remained the language of cultural exchange throughout the nineteenth century. The linguistic hegemony and channels of distribution also favoured popular and more specialized scientific texts. Undoubtedly, journals devoted to systematic reporting and dictionaries benefited the most.

Dictionaries were for several years the direct rivals of academic journals in France—not of periodicals or weeklies devoted to reporting, but of distinguished publications such as the periodicals issued by the Muséum national d'Histoire naturelle or the Académie des sciences. To take one familiar instance, Jean-Baptiste Bory de Saint-Vincent's (1778–1848) sixteen-volume *Dictionnaire classique d'histoire naturelle* found a place in the crammed cabin of the *Beagle* because it contained a considerable number of new descriptions of Australian and Far Eastern species, especially marine invertebrates. In recent years, taxonomists have spent a great deal of work—shaming the historian with their accuracy—in establishing the exact dating of dictionary instalments, to determine the earliest description of species.^32^

Dictionaries, like almost everything else, were issued in instalments. More than a fascination with seriality, the choice was dictated by more mundane reasons. Within Paris, postage for each printed sheet (independently of the folding selected) dispatched within a bound volume cost 5 sous; for an instalment it fell to 2 sous.^33^ Subscription to a periodical, spread over one year, a semester or even a quarter, was economically reasonable, with an exception made for tables and drawings; bound volumes cost much more, for profit but also for distribution reasons. Not to mention the often-remarked point concerning the important savings in printing, acquisition of paper and especially ink: instalments allowed quick adjustments to demand.

For dictionaries, there was the additional advantage of printing and distributing instalments almost at random. An entry from the letter ‘Z’ could well reach subscribers ahead of entries covering the letter ‘A’. What was a financial advantage for the capitalist printer, bookseller or publisher, easily turns into a nightmare for the historian. I mentioned above Cuvier's 1825 article ‘Nature’. Geoffroy responded almost immediately, through his associates at Bory's *Dictionnaire classique*, who included his own son, Isidore. Geoffroy confided his counterblast to the pro-Napoleonic, liberal *Encyclopédie moderne* (23 volumes, 1823–1832) edited by Eustache-Marie Courtin (1769–1839). Geoffroy's article ‘Nature’ is now bound in volume 17, dated 1829. Cogent internal evidence suggests that the politically juicy instalment asking readers how it was possible that Cuvier, the Protestant member of the reactionary Council of State, dared to assess the religious orthodoxy of a good Catholic (Geoffroy himself), was actually written earlier than 1829.

In 1824 Geoffroy had already been attacked at the Académie by the ultra-catholic mathematician Augustin-Louis Cauchy (1759–1857), who doubted Geoffroy was a believer. The naturalist had immediately written to the chancellor of the University of Paris, the powerful ecclesiastic Denis-Luc Frayssinous (1765–1841), reiterating his fidelity to the Church, and got a kindly worded, albeit stern, warning to watch what he said in public. In those charged and dangerous years, when people were easily dismissed from their chairs, to publicly rebut Cuvier and Cauchy was vital for Geoffroy.^34^

Dictionaries varied in ambition and reach. Specialized dictionaries on natural history were running in parallel with more encyclopaedic ventures, or publications such as the fascinating, politically conservative *Dictionnaire de la conversation et de la lecture* (1832–1839, 52 volumes): the entries on scientific topics and biographies of *savants* reveal how important scientific issues were to polite conversation. Some dictionaries even joined forces with periodicals, as part of the same publisher's strategy. Thus, the last of the Panckoucke publishing dynasty, Charles-Louis (1780–1844), could not resist the call of a trade he had learned from the cradle.^35^ From 1811 to 1822 he produced a mammoth *Dictionnaire des sciences médicales* (60 volumes), unjustly neglected by historians, followed by volumes of biographies of medical people and of medical flora.^36^ Most of the key contributors were or had been doctors in the Napoleonic armies, stationed in Germany, where they had enjoyed daily interaction with colleagues from several universities. Panckoucke became a millionaire, and in 1818 he experimented with the periodical as companion to the dictionary, the *Journal complémentaire du Dictionnaire des sciences médicales* we have already alluded to. The journal and the dictionary worked hand in hand, so to speak, using the same staff, often recycling the same editorial contents. Here, the dictionary and the periodical concurred to make the capitalist publisher richer; and they make fascinating reading.

The vocation of dictionaries, however, was not solely or exclusively to give voice to authors and trends ostracized by the academies and excluded from institutional scientific periodicals. Indeed, several prominent academics built their careers on contributions to publishing ventures never or very rarely mentioned in high scientific discourse.^37^ Dictionaries essentially catered for a range of readers spanning the cultivated elites, down to the artisan and commercial class. The multiplication of dictionaries and encyclopaedias is a witness to the profitability and success of the genre. A popular enterprise that started with the promise to stay cheap and short grew to nine thick volumes. It would have been foolish not to fulfil the demand.^38^ At higher social levels, dictionaries made opinion, on science as on everything else. People who would never subscribe to a scientific periodical (whether professional or popular) or to a science-oriented dictionary, found in many generalist dictionaries enough scientific information to engage in conversation in the hundreds of salons active in Paris, now democratized down to gatherings of civil servants, the military, medical personnel, merchants and bankers.^39^

Dictionary instalments were produced and distributed as periodicals were, and were perceived to function as such: they provided information, participated in debate and could be deadly in their venomous criticism or consoling in their praise. They could also be influenced and carefully manoeuvred. By insisting on diachronic categorical developments concerning, in turn and exclusively, periodicals, correspondences, objects, conversations, epistemic objects, iconography or material culture, we risk missing the dense synchronic negotiations between genres and practices involving people and power, real or symbolic. Authors and people appeared to use all available tools that suited them or their social and intellectual ambitions.

The last case to which I wish to call attention concerns once more Geoffroy, a specialist, but not the only one, in pragmatic and opportunistic use of whatever was available to further his plans. In 1836, 10 years after the attack by Cuvier in the dictionary article ‘Nature’, Geoffroy was under respectful fire from Henri Belfield-Lefevre, who accused him of pantheism in a biographical sketch printed in volume 30 of the *Dictionnaire de la conversation*. The author acknowledged that Geoffroy was indeed a true Catholic, albeit blind to the philosophical and religious consequences of his doctrines. Geoffroy lost no time, and obtained permission from the editors of the *Dictionnaire* to answer in a new entry. After the letter ‘G’, ‘H’ was the first option available, so Geoffroy compiled in haste the entry ‘Hérésies panthéistiques’, which appeared in one of the instalments of the following volume.^40^

The article was already printed by 7 November 1836, when Geoffroy deposited a copy on the president's desk during the weekly meeting of the Académie. Following academic practice, he could not read the original text, already printed, and presented a new one, more ambitious, that started with a summary of ‘Hérésies panthéistiques’. Following memoirs that he had been reading since June 1836 on Goethe as a man of science, Geoffroy extolled his own work as the culmination of two centuries of progress in natural knowledge.^41^ Geoffroy's main preoccupation was to mark his own space in the fashionable conservative dictionary, and only as a second move did he ask for the implicit sanction of the Académie, by appearing on the pages of *Comptes rendus*. Whereas colleagues were increasingly resentful of Geoffroy's self-centred outbursts, outside the Académie*,* salons and fashionable intellectuals, such as George Sand (1804–1876) and Honoré de Balzac (1799–1850), sided with him. He was seen as a spirit too free to be shackled by sterile academic conventions.^42^

Most of the dictionaries we have referred to went through two or even three editions, though by the 1840s reissues were simply reprints, increasingly out of date and directed to a lower echelon of the reading public. Which of course did not mean that the era of dictionaries was over: Pierre Larousse's (1817–1875) *Grand Dictionnaire universel du XIX siècle* (15 volumes, 1866–1876) was, and is, a monument to scholarship, and the many dictionaries of medicine put out by publishers such as Baillière or Flammarion remained for several decades standard texts for medical teaching. Outside the growing market for popular science, generalist cultural periodicals, such as the immensely prestigious *Revue des deux Mondes*, continued to carry review articles and lengthy comments by leading naturalists and scientists who would not and could not have published the same texts in institutional academic journals. The publics that wished to make up their own minds on hotly debated issues were still there, using, on the whole, reading rooms and libraries rather than subscribing at individual level.

As I stated at the outset, I am rather inclined to abstain from categorical pronouncements on ‘the periodical’, and wish for more in-depth studies to be carried out, possibly across several European countries. I have also stressed that more attention should be paid to the ‘scientist’ as a member of the reading public, sharing priorities and tools of communication. The specialized academic journal was just one of several venues for communication, and not necessarily the most reliable and effective. The expression ‘communicating science’ often employed in studies of periodicals presupposes well-marked disciplinary and institutional demarcations that were not there before the late-nineteenth century. Academies and scientific societies were keen to maintain the opposite, and referred to each other to close ranks. During the 1830 debate on the decline of science in Britain, natural philosophers quoted the authority of the Académie des sciences in France, and the universal respect the institution was held in, to gain more prestige at home. Their assessment of the scientific scene across the Channel would have surprised and perhaps amused hundreds of individuals there active in a great variety of naturalist pursuits. Many indeed respected the Académie and several did their best to join its ranks. Yet, the great majority of French scientific personnel did not belong to the Académie and managed to survive, and at times prosper, as members of the cultivated elites. Many found employment in the arduous market of ideas and narratives about nature: joining ranks with the ‘hommes de lettres’ in filling endless columns in endless editorial ventures dealing with all possible subjects. To them, periodicals, monographs, travel narratives (rigidly sold in instalments) or dictionaries answered basically the same professional, if not survival, needs. Those who proved successful had to be given consideration even by the stars of the academic system. The final contemporary meaning of each tool of communication, including periodicals, was not inscribed in their pages, but in the contextual codes and practices permeating the complex social stratigraphy of writers and readers.

## Notes

1 The instability of such categories is, of course, a key point of this paper. Offprints, for example, have been known by many names at various periods, with the more modern form only ‘taking’ relatively late in the nineteenth century. I use offprint as a generic term here to avoid confusion with separate copies that could sometimes be printed with their own numbering, title page and even year of publication.

2 James E. McClellan III, ‘The scientific press in transition: Rozier's journal and the scientific societies in the 1770s’, *Ann. Sci.* **36**, 425–449 (1979); Peter W. J. Bartrip, *Mirror of medicine: a history of the* British Medical Journal (Clarendon Press, Oxford, 1990); Melinda Baldwin, *Making* Nature*: the history of a scientific journal* (University of Chicago Press, 2015).

3 A. Fyfe, ‘Journals, learned societies and money: *Philosophical Transactions*, ca. 1750–1900’, *Notes Rec.* **69**, 277–299 (2015). See also Sally Shuttleworth and Berris Charnley, this issue.

4 Amongst many others, James A. Secord, ‘Knowledge in transit’, *Isis* **95**, 654–672 (2004); Geoffrey Cantor *et al.*, *Science in the nineteenth-century periodical: reading the magazine of nature* (Cambridge University Press, 2004). I found A. Csiszar, ‘Seriality and the search for order: scientific print and its problems during the late nineteenth century’, *Hist. Sci.* **48**, 399–434 (2010), particularly stimulating.

5 Jeanne Peiffer, Maria Conforti and Patrizia Delpiano (eds), *Scholarly journals in early modern Europe: communication and the construction of knowledge*, special issue, *Arch. inter. d'hist. des sc.*, fasc. 170–171, **63** (June 2013).

6 Peiffer *et al.*, *op. cit*. (note 5), p. 23.

7 Graham Law and Robert L. Patten, ‘The serial revolution’, in *The Cambridge history of the book in Britain*, *Vol. 6, 1830–1914* (ed. David McKitterick), pp. 144–171 (Cambridge University Press, 2014); Nick Hopwood, Simon Schaffer and James A. Secord, ‘Seriality and scientific objects in the nineteenth century’, *Hist. Sci.* **48**, 251–285 (2010).

8 Brenda Dooley (ed.), *The dissemination of news and the emergence of contemporaneity in early modern Europe* (Ashgate, Farnham, 2010); Will Slauter, ‘Le paragraphe mobile’, *Annales* **67**, 2, 253–278 (2012).

9 Jonathan R. Topham, ‘Science, print and crossing borders: importing French science books into Britain, 1789–1815’, in *Geographies of nineteenth-century science* (ed. David N. Livingstone and Charles Withers), pp. 311–44 (University of Chicago Press, 2011).

10 ‘Advertisement’, non-paginated preface, dated 30 June, *Phil. Mag.*, **43** (1814).

11 David M. Bickerton, *Marc-Auguste and Charles Pictet, the* Bibliothèque Britannique (*1796–1815*) *and the dissemination of British literature and science on the Continent* (Slatkine, Geneva, 1986). Réné Sigrist has posted fascinating new letters between Pictet and J.-C. Delamétherie, concerning the launching of the *Bibliothèque Britannique*, *Correspondance de Marc-Auguste Pictet. Partie scientifique et technique*, at Academia.edu http://www.academia.edu/21539717/Correspondance_de_Marc-Auguste_Pictet_1752-1825_._Partie_scientifique_et_technique_Tome_IVb_Les_correspondants_italiens_allemands_et_autres_version_mise_%C3%A0_jour (accessed 25 August 2016).

12 Antoine Lilty, *The world of salons: sociability and worldliness in eighteenth-century Paris* (Oxford University Press, 2015); Roger Chartier, *The cultural origins of the French Revolution* (Duke University Press, 1991), and *The order of books: readers, authors and libraries in Europe between the 14th and 18th centuries* (Stanford University Press, 1994). See Jean-Luc Chappey, *La Société des Observateurs de l'Homme (1799–1804). Des anthropologues au temps de Bonaparte* (Société des Études Robespierristes, Paris, 2002) for a penetrating analysis of the varied population writing about natural history in the aftermath of the Revolution.

13 Stendhal, ‘Sketches of Parisian life’, *New Month. Mag.* **16**, 508–518 (1826) and ‘Letters from Paris by Grimm's grandson. N. III’, *London Mag.* **1**, 339–347 (1825).

14 Stendhal, ‘Letters from Paris by Grimm's grandson. N. IX’, *London Mag.* **3**, 130–132 (1825), at p. 131. Stendhal poked fun at the Académie des sciences and Laplace's baseness to power, and noted that Geoffroy Saint-Hilaire was now emerging as a threat to Cuvier.

15 Stendhal, ‘Sketches of Parisian life’, *New Month. Mag.* **23**, 188–191 (1828). Jesuits, Stendhal reported, estimated that over two million copies of Voltaire's works had been published over the previous 10 years in scores of editions, two-thirds of which went to the provinces, p. 188.

16 Stendhal, ‘Sketches of Parisian life’, *New Month. Mag.* **17**, 284–288 (1826), at p. 285 (on ‘the tribunal of public opinion’).

17 Jean-Paul Marat*, Recherches physiques sur le feu* (Jombert, Paris, 1780) and *Recherches physiques sur l’électricité* (Clousier, Paris, 1782); Bernard Germain Étienne de Laville-sur-Illon, comte de Lacépède, *Essai sur l'électricité naturelle et artificielle* (Didot, Paris, 1781) and *Physique générale* (Didot, Paris, 1782–1784).

18 Maria Conforti, ‘Medicine and life sciences in learned journals in Naples between the end of the Ancien Régime and the French decade’, in Peiffer *et al.*, *op. cit*. (note 5), pp. 455–474, 458.

19 Louis Eugène Hatin, *Bibliographie historique et critique de la presse périodique française* (Firmin-Didot, Paris, 1866).

20 Thomas Broman, ‘Expertise without experts: anonymity and medical authority in Johann August Unzer's *Der Artze*’, in Peiffer *et al.*, *op. cit*. (note 5), pp. 29–47, has discussed the crucial difference between the authority claim of ‘academic’ publications and the ‘friendly’ complicity some commercial ventures established with their readers.

21 Roger Cooter and Stephen Pumfrey, ‘Separate spheres and public places: reflections on the history of science popularization and science in popular culture’, *Hist. Sci.* **32**, 237–267 (1994); Bernard Lightman, *Victorian popularizers of science: designing nature for new audiences* (University of Chicago Press, 2010); Geoffrey Cantor and Sally Shuttleworth (eds), *Science serialized: representations of the sciences in nineteenth-century periodicals* (MIT Press, Cambridge, MA, 2004); and Louise Henson, Geoffrey Cantor, Richard Noakes, Sally Shuttleworth, Gowan Dawson and Jonathan R. Topham (eds), *Culture and science in the nineteenth-century media* (Ashgate, Aldershot, 2004).

22 Richard D. Whitley, ‘Knowledge producers and knowledge acquirers: popularization as a relation between scientific fields and their production’, in *Expository science: forms and functions of popularisation* (ed. Terry Shinn and Richard D. Whitley), pp. 3–28 (Reidel, Dordrecht and Boston, 1985).

23 An anonymous referee has pointed out to me that n. XL of the *Edinburgh New Philosophical Journal* containing the article on Geoffroy was advertised for sale in the *Standard* on 15 April 1829.

24 For the relevant bibliography see William Jenkins, ‘New perspectives on Edinburgh Lamarckians and other transformist thinkers: evolutionary debates in the Athens of the North, 1790–1844’, PhD dissertation, University of Edinburgh (2015)*.*

25 ‘Sciences. Académie des sciences. Séance du lundi 24 mars 1829. De la continuité du règne animal, par voie de génération, depuis les premiers âges du monde jusqu'à nos jours', *Le Globe*, **7**, 26, 207–208 (1 April 1829).

26 *L'Observateur médical* **6**, 176–180 (1829).

27 See instances of this in Pietro Corsi, *Fossils and reputations: a scientific correspondence, Pisa, Paris, London, 1854–1857* (Pisa University Press, 2008).

28 Corsi, *op. cit*. (note 27); ‘Much ado about nothing: the Italian Geological Survey, 1861–2006’, in ‘Thematic set of papers on Geological Surveys’ (ed. Pietro Corsi) in *Earth Sci. Hist.* **26**, 97–125 (2007), and ‘The Italian Geological Survey: the early history of a divided community’, in *Four centuries of the word ‘Geology’* (ed. Giovanni Battista Vai and Walter Cavazza), pp. 255–279 (Minerva Edizioni, Bologna, 2003). For the progress of the *Bollettino* as recorded in the pages of the Annual Report of the Survey, see http://histmap.huma-num.fr/presentation-it.php?lang=en (accessed 16 June 2016).

29 Fabiano Ardigó (ed.), *Histórias de uma ciência regional: cientistas e suas instituições no Paraná (1940–1960)* (Contexto, São Paulo, 2011).

30 Georges Cuvier, ‘Nature’, *Dictionnaire des sciences naturelles* **34**, 261–268 (1825).

31 I have discussed several phases of the war for the control of cultivated public opinion in *The age of Lamarck: evolutionary theories in France, 1790–1830* (University of California Press*,* 1988).

32 Neal L. Evenhuis, ‘Dating, publication and authorship of Froriep's *Wörterbuch der Naturgeschichte* (1824–1837)’, *Bishop Mus. Bull. in Zool.* **9**, 49–58 (2015), for a listing of his many important contributions covering dictionary as well as periodical instalment dating.

33 André Cabanis, *La presse sous le Consulat et l'Empire (1799–1814)* (Société d’études Robespierristes, Paris, 1976), p. 133. For the British Isles, see Law and Patten, *op. cit.* (note 7).

34 Étienne Geoffroy Saint-Hilaire, letter to Frayssinous dated 20 July 1824, and answer dated 23 August, in Archives Nationales, Pierrefitte-sur-Seine, F/17/20824/B. Cauchy attacked Geoffroy on 12 July 1824 in writing, see *Bull. Gén. et univ. Sci. médicales*, section **3**, 177 (1824). The President of the Académie rejected Cauchy's text because it contained speculations on the religious beliefs of a colleague.

35 His older sister, Antoinette-Pauline Agasse (1769–1843), known as ‘Veuve Agasse’ after the death of Henri Agasse (1752–1813). Henri had bought the Panckoucke empire in January 1794 to save it from bankruptcy. ‘Veuve Agasse’ was running the privately owned, yet official, daily of the Revolution and the Empire, the *Moniteur universel*.

36 *Biographie médicale*, 7 vols (1820–1825); *Dictionnaire abrégé des sciences médicales*, 15 vols (1821–1826). A literary figure such as Stendhal was well acquainted with the *Dictionnaire*, ‘Sketches of Parisian life’, *New Month. Mag.* **16**, 200 (1826).

37 Isidore Geoffroy Saint-Hilaire collected his many contributions to dictionaries in his influential *Essais de zoologie générale* (Roret, Paris, 1841).

38 Félix-Édouard Guérin-Meneville, *Dictionnaire pittoresque d'histoire naturelle*, 9 vols (1833–1839). Guérin was also the editor of the successful *Gazette médicale*, reporting on academic controversies.

39 Jean-Jacques Coulmann, *Réminiscences*, 3 vols (M. Levy, Paris, 1862–1869). Coulmann, a relative of Cuvier, has left a fascinating description of middle-class salon sociability during the 1820s and 1830s.

40 I have not gone through the lengthy process of establishing when the instalment containing the entry in question did appear as part of the dictionary.

41 Henri Belfield-Lefevre, ‘Geoffroy Saint-Hilaire’, *Dictionnaire de la conversation* **30**, 121–126 (1836). The instalment was released in March 1836, as reported in *Bibliographie de la France* **25**, 159 (2 April 1836). Étienne Geoffroy Saint-Hilaire, ‘Hérésies panthéistiques’, *Dictionnaire de la conversation* **31**, 384–389 (1836); *Comptes-rendus hebd. Séan. Acad. Sci.* **3**, 523–527 (July–December 1836). See also *La Presse* **128**, 3 (17 November 1836) for Geoffroy defending himself in front of his Sorbonne students.

42 Pietro Corsi, ‘The revolutions of evolution: Geoffroy and Lamarck, 1825–1840’, *Bull. du Musée d'Anth. Préhist. de Monaco* **51**, 97–122 (2011).

